# Lessons learned from pandemic response to COVID-19 in Bangladesh: NGO-based emergency response framework for low- and middle-income countries

**DOI:** 10.1186/s12913-023-09643-w

**Published:** 2023-06-20

**Authors:** Tanvir Ahmed, Parsa Musarrat, Zarina Nahar Kabir

**Affiliations:** 1 Centre for Clinical Research and Health Innovation, SAJIDA Foundation, Dhaka, Bangladesh; 2 Department of Research, SAJIDA Foundation, Dhaka, Bangladesh; 3 Department of Neurobiology, Care Sciences and Society, Karolinska Institute, Stockholm, Sweden

**Keywords:** Low- and middle-income countries (LMICs), Emergency response, Pandemic response, Bangladesh, Non-Government Organisation (NGO)

## Abstract

**Background:**

Response to COVID-19 pandemic in Bangladesh was led by the Government of Bangladesh aided by Non-Government Organisations (NGO) among others. The aim of the study was to explore the activities of such an NGO to understand the philosophy, aspiration and strategy to plan and implement an effective response to COVID-19 pandemic in Bangladesh.

**Methods:**

A case study of a Bangladeshi NGO called SAJIDA Foundation (SF) is presented. From September to November 2021, using document review, field observation and in-depth interviews, four aspects of their COVID-19 pandemic related activities was explored – a) why and how SF initiated their COVID response; b) what adaptations were made to their usual programmes; c) how SF’s response to COVID-19 were designed and what were the anticipated challenges including overcoming measures; and d) perception of the staff about SF’s activities related to COVID-19. Fifteen in-depth interviews were conducted with three groups of SF staff: *frontliners, managers* and *leaders.*

**Result:**

The impact of COVID-19 has been beyond health emergencies and posed multidimensional challenges. SF took a two-pronged approach – aid the government to respond to the emergency and adopt an all-inclusive plan to address diverse challenges related to overall well-being of the population. The underlying strategy of their response has been to: define the challenge of COVID-19 and identify required expertise and resources, ensure people’s health and social wellbeing, adjust existing organisational processes, ensure functional partnership with other organisations for effective resource and task sharing, and safeguard health and wellbeing of the organisation’s own employees.

**Conclusion:**

The findings suggest a ‘4C framework’ including four components as the basis of a comprehensive response to emergencies by NGOs: 1. *Capability assessment* to identify *who* are in need and *what* is needed; 2. *Collaboration* with stakeholders to pool resources and expertise; 3. *Compassionate leadership* to ensure health and social safety of the employees which ensures their dedication in managing the emergency; and 4. *Communication* for quick and effective decision making, decentralisation, monitoring and coordination. It is expected that this ‘4C framework’ can help NGOs to embark on a comprehensive response to manage emergencies in resource constrained low- and middle-income countries.

## Background

Resource scarcity and myriad of pre-existing socioeconomic and health-related challenges resulted in the burden of the COVID-19 (coronavirus disease-19) pandemic being heavier on low- and middle-income countries (LMICs) compared to high income countries (HICs). Evidence suggests that COVID-related mortality in poorer countries have been four times higher than the richer countries [1] The socioeconomic and health-related impact of COVID has been described to be ‘deadly’ for the LMICs [2, 3]. While the primary focus of the LMICs has been on controlling the infection, long periods of lockdown(s) essentially brought a complete stop to daily lives affecting specially those working in informal sectors and labour-intensive industries [4, 5]. Thus, at one hand, there were challenges of allocating additional resources for emergency provision of COVID-19-related healthcare (i.e., setting up screening facilities and dedicated medical wards, expanding intensive care units, etc.) and mass communication for behavioural change. On the other hand, there were challenges in keeping the economic and other usual health and development activities ongoing. As a result, the capacity of the government of LMICs was stretched beyond the limits and Non-Government Organisations (NGOs) played crucial role in aiding the governments to respond to the COVID-19 pandemic. Bangladesh too faced such two-pronged challenges and many for- and not-for-profit private sectors including NGOs played notable role in responding to the pandemic.

### Bangladesh and COVID-19

On 11 March 2020, WHO declared COVID-19 a global pandemic [6, 7]. As of March 29, 2023 there have been 2,038,014 confirmed cases of COVID-19 with 29,446 deaths [8]. As soon as the first case was identified in early March 2022, the Government of Bangladesh published guidelines for infection prevention and later closing down of institutions began starting with all educational institutions [9, 10]. This was followed by strict lockdown, use of personal protective equipment (PPE) by healthcare staff, practice of social distancing in public places, frequent handwashing, use of face mask, suspension of air travel, closing down most factories, etc. [11, 12]. The Ministry of Labour and Employment of Bangladesh reported that more than two million ready-made garment (RMG) workers were unemployed by the end of March 2020 [13]. Evidence suggests that the economic impact of COVID-19 in the country resulted in 24.5 million ‘new poor’ being pushed into poverty [14, 15]. While this is a concern for widespread mental health issues [5, 16], recent evidence suggests that COVID-19 pandemic affected women more than men in terms of mental health such as anxiety, depression and stress [16]. Also, domestic violence against women increased during the pandemic [17]. As for demand on healthcare, many patients who were hospitalized due to COVID-19 required intensive care. In 2018, Bangladesh had a total of 5,206 hospitals, of which 610 were public and 4,596 private [18]. Out of the 610 government hospitals, only 27 had ICUs (intensive care unit) with general to ICU bed ratio of 219:1, whereas the standard should be 10:1 [4]. While 78% were in the private sector, access to ICUs remained elusive to most people due to high costs [18]. As a result, government hospitals were overwhelmed due to high demand coupled with limited knowledge of the disease and its treatment. In addition, due to lockdown and lack of availability of PPEs, other essential and existing public health issues and related service provisions started declining rapidly [10].

It is important to note that, Bangladesh is a country with resource scarcity and challenges of access to just and quality healthcare. Because of the multidimensional challanges brought on by the COVID-19 (snapshot of which is presented above), the impact of the pandemic thus has been two pronged for Bangladesh.

### Initial response by NGOs to COVID-19 in Bangladesh

Historically, Bangladesh has had many local and international NGOs to address various development issues. This continued during the pandemic as well. While the Government of Bangladesh responded to the pandemic as early as the middle of March 2020, the local and international NGOs too started responding to the challenges of pandemic within their own capacity. Activities were largely focused on gathering funds, raising awareness and support the poor household by providing food, financial aid, etc. [11, 12]. These responses by the NGOs have largely been focused on their area of work and targeted population rather than an organised effort aimed at strengthening national response.

SAJIDA Foundation (SF) is an NGO in Bangladesh working towards bringing sustainable social and economic changes in the country since 1993 and has been active in addressing the challenges of the pandemic. With experience of implementing multi-faceted programmes, SF designed new programmes and adapted existing ones to cater to the social and economic needs brought about by the pandemic. SF recognized the need to work, not just parallelly but under the government’s stewardship as a response to the pandemic. The objective of this case study was to document the multidimensional response to COVID-19 by SAJIDA Foundation, and understand the philosophy, aspiration and strategy behind the organisation’s response to the pandemic. Given the scarcity of resources, limited capacity of the government and role of NGOs, the findings can be useful in devising organisational strategy for a concerted pandemic response and related mitigation plan in Bangladesh and other relevant LMIC contexts in the future.

## Methods

### Design

The chosen design of this study was ‘case study,’ a pragmatic approach to understand explicit and implicit links and pathways of implementation of an event or phenomena as it occurs through blending of multiple methods [19].

### Data collection period, tools and techniques, and study participants

Due to the fast changing response to challenges posed by COVID-19, data collection period of this study was kept focused and short. Data for this study was collected in three steps, from September 01 to November 30, 2021. The first step was to make a list of all activities of SF in relation to its response to COVID-19 over a period of 2020 to 2022. For this, review of related organisational documents (both print and online) (Table 1), and informal discussions with relevant staff was done. During the time of this study, SF was working in two of the bordering districts to set up COVID-19 dedicated medical and ICU wards at the corresponding district hospitals. The second step was to visit two district hospitals, Jashore and Meherpur, to observe how SF was helping in setting up the wards for patients of COVID-19. This helped in understanding how SF was collaborating with the Government of Bangladesh in rendering COVID-19 related healthcare, what and how services were being rendered and response of the community and government staff at the hospital towards SF’s work.

**Table 1:** List of reviewed organisational documents

Organisational Reports: • 2019-2020 Annual Report, SF (Annual-Report-2020.pdf (sajida.org)) • 2021 Annual Report, SF (Annual-Report-2021.pdf (sajida.org)) • Progress Report: Mask Making & Tailoring Training with Swift Earning Opportunities • Cumulative Summary Report for Web Portal (COVID-19 Pandemic Response) • SF MIS Data	
Website: • SAJIDA website: COVID-19 Initiatives Web Portal • Lost Stock website: Helping Workers – SAJIDA Foundation, Lost Stock (https://loststock.com/pages/helping-workers)	
SF Newsroom: • SAJIDA Distributes Thousands of Leaflets to Create Awareness on COVID-19 (Sajida distributes thousands of Leftlets to create awareness on COVID-19 | Sajida foundation | Newsroom) • SAJIDA Has Continued to Disburse Food Vouchers to Vulnerable Communities (Sajida has continued to disburse food vouchers to vulnerable communities | Sajida foundation | Newsroom) • SAJIDA Foundation Has Successfully Distributed Food and Hygiene Packages in Dhaka South City Corporation (Sajida foundation has successfully distributed food and hygiene packages in dhaka south city corporation | Sajida foundation | Newsroom) • SAJIDA Foundation Distributed 12,000 Food & Hygiene Packs to the Destitute and Low-Income Families (Sajida foundation distributed 12,000 food & hygiene packs to the destitute and lowincome families | Sajida foundation | Newsroom) • SAJIDA Hospital, Keraniganj Opened its New COVID-19 Treatment Unit (Sajida hospital, keraniganj opened its new covid19 treatment unit | Sajida foundation | Newsroom) • SAJIDA Signed an MoU to Deploy a Team of Expert Health Professionals at 250-Bed General Hospital, Jashore (Sajida signed an mou to deploy a team of expert health professionals at 250-bed general hospital, jashore | Sajida foundation | Newsroom) • SAJIDA Hospital Has Expanded its Services by Assisting Four Public Hospitals at Jashore, Chuadanga, Pabna and Meherpur (Sajida hospital has expanded its services by assisting four public hospitals at jashore, chuadanga, pabna and meherpur | Sajida foundation | Newsroom) • Inauguration of Cash Grant Support to the Urban Extreme Poor by Proshomon (Inauguration of cash grant support to the urban extreme poor by proshomon | Sajida foundation | Newsroom) • SAJIDA Foundation’s Agriculture Unit Stepped Up and Helped the Farmers (Sajida foundation’s agriculture unit stepped up and helped the farmers | Sajida foundation | Newsroom) • Approx. 5,000 RMG Workers Will be Registered by SAJIDA For Receiving COVID-19 Vaccine (Approx. 5,000 rmg workers will be registered by sajida for receiving covid-19 vaccine | Sajida foundation | Newsroom) • Pariticpants for COVID-19 Vaccination in Maniknagar, Dhaka (Amrao manush program has initiated registering program participants for covid-19 vaccination in maniknagar, dhaka | Sajida foundation | Newsroom) • Supporting the ReadyMade Garments Industry and its Workers With “Lost Stock” Clothing Box (Supporting the readymade garments industry and its workers with “lost stock” clothing box • | Sajida foundation | Newsroom) • Mask Making & Sewing Training With Rapid Learning Opportunities – A Timely Project By SAJIDA Foundation (mask making & sewing training with rapid learning opportunities” – a timely project by sajida foundation | Sajida foundation | Newsroom)	

The final step was to conduct a series of interviews to understand the aspiration, motivation and organisational approach which altogether constituted SF’s response to the COVID-19 pandemic. These interviews were done using an open-ended guideline using four dimensions as themes: a) why and how SF initiated its response to the COVID-19 pandemic, b) changes/adaptations made to its usual activities/care provision of SF (if any), c) how SF’s COVID-19 response was designed, what challenges were anticipated and how those were overcome, and d) employee perception of SF’s COVID-19 related activities. Altogether 15 interviews were conducted with three groups of key-informant (all SF employees): a) those who implemented these activities, i.e., *the frontliners;* b) those who coordinated these activities, *the managers* and c) senior staff who provided leadership and guidance, *the leaders.* Table 2 lists the key informants who were mostly chosen through review of documents and identifying people who implemented and/or led the COVID-19 related activities of SF. Others were chosen based on suggestions by some of the key informants due on their key roles in the response. All interviews were conducted using online platform (Microsoft Teams) to ensure physical distancing, except one which was conducted face to face maintaining all safety measures. The length of the interviews was about an hour and was conducted by TA and two trained research assistants.

**Table 2:** List of key informants

Frontliners (who implemented SF’s COVID related activities)
1. Customer Care Officer, SF district ICU team, Meherpur
2. Medical Officer, SF district ICU team, Meherpur
3. Nursing Supervisor, SF district ICU team, Meherpur
4. Emergency Medical Officer, SF Narayanganj COVID Hospital
5. Medical Officer, SF Narayanganj COVID Hospital
6. Assistant General Manager, SF Keraniganj Hospital
7. Clinic Manager, SF Narayanganj COVID Hospital
8. Medical Officer (Remote), Home Isolation Management and Community Mobilization (HIMCM) Project
Managers (who coordinated SF’s COVID related activities)
9. Chief Operating Officer, Home and Community Care Ltd. (HCCL), SF
10. Senior Coordinator, Development Programs, SF
11. Senior Coordinator, Water, Sanitation and Hygiene (WASH) Programme, SF
12. Coordinator, Development Program, SF
Leaders (senior staff who provided leadership and guidance towards SF’s COVID related activities)
13. Chief Executive Officer, SF
14. Senior Director—Head of Hospital (Health Programme), SF
15. Deputy Executive Director, SF

### Data management and analysis

Data of this study comes from review of documents, online information, informal discussions, observations and in-depth interviews. The main aim of the review of the documents and online information was to prepare a list of SF’s COVID-19 related activities and identify the frontliners, managers and leaders.

The organisational reports, online and printed materials related to COVID-19 were first reviewed to prepare a timeline to portray the COVID-19 related response by SF. All interviews with the key informants were recorded. As soon as the interviews ended, a summary was prepared to document interview notes. All recorded interviews were transcribed verbatim. The transcripts were then read independently by members of the research team and thereafter, codes, categories and themes were identified. The categories and themes were then organised axiomatically to identify typical and atypical pattern(s), which with appropriate contextualization, has been reported as findings.

## Results

As mentioned in the methods section, the data of this study was collected in three steps – 1. A list was created of all COVID-related activities by SF through review of organisational documents and informal discussions with staff; 2. Observation technique was employed to obtain an idea of how some of those activities were implemented in real life; and 3. A series of interviews were conducted with key informants to understand the underlying organisational aspiration, motivation and organisational approach behind these activities. These steps helped in analysis by blending the data obtained from the three sources and thus the following subsections present data obtained from more than one source*.*

### Snapshot of SF’s COVID-19 response activities

From March 2020 to February 2022, SF undertook a range of activities which altogether constituted its response to COVID-19 pandemic in Bangladesh. These activities were either newly designed/devised or adapted/added to existing development (including health) activities to address the impending threat of the pandemic. These activities were targeted to the community at large, certain groups ranging from poor households or children with special needs, and/or people with COVID-19 like symptoms, etc. Thus, SF’s COVID-19 pandemic related activities can be grouped as provision of health services, infection prevention and risk reduction services for the community and SF frontline staff, and social support for the vulnerable households to help continue with daily living. One of the members of the SF leadership and a key-informant of this study mentioned that *“Our vision is health, happiness and dignity. All of our COVID-19 related initiatives were in line with our vision. Our mission is ensuring quality of life by implementing sustainable effective interventions.”*

Provision of health services:

- During COVID-19 pandemic, SF was among the first one to respond to ensure access to required healthcare in Bangladesh alongside the government. One of the key-informant from the SAJIDA hospital, Narayanganj mentioned,

*“SAJIDA Foundation has always worked for the need of the country during emergency. For instance, if there’s a fire in a slum, SAJIDA Foundation is there. When Rana Plaza accident (a garment which collapsed in 2013 in Bangladesh) happened, SAJIDA Foundation was there. And when COVID came, SAJIDA Foundation decided to take part in COVID-19 health response.”*

These services were provided to help people who were COVID-19 positive or had symptoms similar to COVID-19. The services were also extended to anyone who needed access to general healthcare which was in decline due to ongoing COVID-19 waves. The services included hospital-based care, preliminary check up by paramedics by setting up kiosks, arranging for polymerase chain reaction (PCR) tests to diagnose COVID-19, setting up telemedicine services open to all, mental health consultation for the general population and SF employees, online and in person therapy for children with special needs, and strengthening domiciliary services for older persons to help cope with the risks of COVID-19 and their existing morbidities. Table 3 lists the health services that were provided by SF as part of their response to the COVID-19 pandemic.

**Table 3:** SF’s COVID-19 response: provision of health services

Activities	Start	End
*Provision of direct health services*		
COVID-19 Hospital Support and Treatment (SF Hospitals)	March 2020	February 2022
*SAJIDA Hospital Narayanganj (dedicated to patients of COVID-19 only)*		
Set up COVID unit with ICU and general ward, sending 1,746 samples for RT-PCR test, training of the hospital staff members through the government’s training programme	March 2020	January 2021
Convalescent plasma therapies carried out for COVID-19 patients	June 2020	-
All hospital beds converted into semi-critical ones. Quality control personnel & certified dietician appointed	July 2020	-
Bed capacity increased from 50 to 60	August 2020	-
Adding ventilator and three-ball spirometer to the hospital inventory	October 2020	-
*SAJIDA Hospital Keraniganj*		
Primary check-up kiosks set outside the hospital, operated by paramedics for visitors	June 2020	June 2022
Setting up COVID unit with 6-bed ICU and 22 semi-critical bed, sending 224 samples for RT-PCR test	January 2021	February 2022
*COVID-19 Hospital Support and Treatment (Collaboration with Government Hospitals in Border Districts)*		
Co-establish COVID unit with 11 bed ICU and 20 bed ward at the District (Government) Hospital, Jessore, a 250-bed secondary care public hospital	June 2021	October 2021
Co-establish COVID unit with 6 bed ICU and 8 bed ward at the District (Government) Hospital, Chuadanga, a 250-bed secondary care public hospital	July 2021	September 2021
Co-establish COVID unit with 2-bed COVID ward at the 250-bed General (Government) Hospital, Pabna	August 2021	September 2021
Co-establish COVID unit with 5 bed ICU and 6 bed ward at the District (Government) Hospital, Meherpur, a 250-bed secondary care public hospital	August 2021	October 2021
Telemedicine		
Hotline Service for on-call consultation: served 6,230 people and spread health and hygiene awareness message to 579,570 clients	March 2020	March 2022
COVID Symptom Checker Messenger Chatbot: reached 10,711 clients	April 2020	June 2022
SAJIDA Mental Health Programme and Psychological Health and Wellness Centre		
Phone and video counselling sessions	April 2020	Ongoing
Tele-counselling service to provide on-call emotional support (in collaboration with BRAC and *Kaan Pete Roi’*)	June 2020	December 2020
Employee assistance service and webinars for mental health support	September 2020	Ongoing
Home and Community Care Limited (HCCL)		
Stay-in caregiver provider for older persons	April 2020	Ongoing
Home doctor visit and physiotherapy for senior citizens and home care for mild cases of COVID-19	August 2020	Ongoing
Isolation centres for HCCL caregivers	October 2020	May 2022
Home testing facilities for older persons	September 2020	Ongoing
Care for Children with Special Needs – Inner Circle		
Online therapy sessions for children with special needs	April 2020	June 2021
Restart of physiotherapy sessions	July 2021	Ongoing
*Provision of infection prevention and risk reduction services for the community and SF frontline staff*		
Hygiene Behaviour Promotion		
Water, Sanitation and Hygiene (WASH) programme installed 585 pedal-based handwashing devices in urban poor areas and its is estimated that about 2,289,732 people have been benefited by these handwashing devices	March 2020	-
About 50,000 leaflets were distributed to extreme poor households of urban slams of Dhaka city	March 2020	May 2021
One disinfection tunnel installed by WASH at SF Narayanganj Hospital	May 2020	-
In total, 30 slums in DSCC were decontaminated which covered 49,877 people	May 2020	May 2021
Hygiene Pack Distribution		
Total 316,232 individuals received hygiene kits	June 2020	February 2022
Personal Protective Equipment (PPE) Distribution		
Altogether 11,000 PPE sets and 56,140 PPE distributed among hospital staff and home care workers (HCCL)	March 2020	-
Total 450 sets of PPEs were distributed to the empanelled hospitals in Feni and Chandpur districts by PROSHOMON (one of the urban based health programmes of SF)	March 2020	-
Pneumask distributed among SF Narayanganj Hospital staff, along with 22,600 surgical masks and 6,753 cotton masks	November 2020	-
Total 7,000 masks were distributed among members of SF’s Water Credit programme	December 2020	-
Vaccine Registration Support		
WASH helped 4,919 beneficiaries, including ready-made garment (RMG) workers and members in South Dhaka to register for vaccination	March 2021	October 2021
*Amrao Manush (we too are human)* (also known as Improving Livelihoods of Urban Extreme Poor-Pavement Dwellers Centre supported 1,363 beneficiaries for vaccination registration	March 2021	February 2022
Urban Extreme Poor Programme supported 929 beneficiaries for vaccination registration	July 2021	Ongoing

As Table 3 shows, hospital-based services have been the core of SF’s COVID-19 response. As the COVID-19 cases started rising, the need for trained healthcare staff and access to general and advanced centre-based COVID care, especially for the lower income groups, became essential. This led SF to immediately set up a hospital dedicated to patients with COVID-19 in a city called Narayanganj, adjacent to the capital Dhaka. It is a popular industrial district in the country with a large proportion of the inhabitants belonging to lower income population group. Similar set up was established in SF’s hospital in Keraniganj, a subdistrict of Dhaka. This hospital was established to ensure access to quality healthcare for the low-income group who constitutes a significant share of Keraniganj’s population. Later in 2020, as rates of COVID-19 infection started rising in other districts (especially the border districts), SF reached out to the district hospitals and local administration – primarily in areas with high number of COVID-19 positive cases. Four (Jashore, Chuadanga, Pabna and Meherpur) districts responded and SF strengthened the capacity of the corresponding district (government) hospitals to provide care for patients with COVID-19. In these four public hospitals, SF helped in setting up COVID-19 units and made provisions for necessary equipment (e.g., ventilators and dialysis machines). SF also helped redesign the COVID area in the hospitals to include a triage area, separate walkways for patients and staff, and set up general wards and advanced wards such as intensive care, high dependency and semi-critical units.

Prevention of COVID-19 has been SF’s focus from the very beginning of its response. Thus, part of SF’s COVID-19 related activities included infection prevention and risk reduction services for the community and SF frontline staff. Before COVID-19 was first detected in Bangladesh, SF had already taken initiative to spread awareness about the disease through social media. In March 2020, SF began distributing leaflets in urban slums of Dhaka city on ‘Do’s and Don’ts’ in relation to personal hygiene – including social distancing, coughing/sneezing etiquette, etc. – to reduce chances of transmission and importance of handwashing. Such awareness initiatives also included multiple seminars and webinars to spread the message. To enable people to practice hygiene behaviour, at a later stage, SF distributed hygiene kits containing soap, detergent and sanitizers, and installed a number of portable pedal-based handwashing devices, primarily in urban slums and other SF programme areas. To prevent infection, SF also participated in aiding the government’s vaccine programme by helping their beneficiaries to register and complete the process of getting the vaccine. To help employees who were working in the frontline, SF distributed and mandated use of PPEs (for the healthcare staff) and face masks (for others). Table 3 includes the infection prevention and risk reduction activities conducted by SF as part of its COVID response.

Social support for the vulnerable households to help continue with daily living:

- As the pandemic unfolded, alike other countries, Bangladeshis too experienced hindrances in living a normal life, which affected the poorer households most. This led SF’s COVID-19 response to consider and implement a wide range of social support activities from the very early days of the pandemic in Bangladesh. One of the key-informants among the leader group, mentioned that – *“Our CEO always thinks critically. Not just COVID but in any situation, she thinks critically and tries to look ahead and ask – what may happen next?”* Accordingly, in May 2020 (about two months after the identification of the first case in the country), SF launched a Food Aid project and eventually, SF’s other programmes also distributed food packages among their beneficiaries – which consisted of rice, potatoes, salt and oil – among poor households in urban slums, RMG workers and other minority groups. SF’s education programme, providing formal, informal and vocational education to destitute children, adopted phone call based distant teaching. For the domestic workers who lost their jobs, SF launched a project on mask making and sewing training with rapid earning opportunities. Along with these skills, they were given financial support and equipment. SF also devised a market linkage programme whereby SF acted as an intermediary for farmers to sell their produce during the lockdown and provided delivery service. Most farmers who accessed this programme were members of SF’s microfinance programme. In addition, SF provided financial support to beneficiaries through their microfinance and other programmes. In July 2020, the microfinance programme rescheduled their loan schemes with flexible repayment options and waived service charges for their members. Water, Sanitation and Hygiene (WASH) programme arranged for loans called ‘Water Credit’ for their members to help build sanitation facilities during the pandemic. Table 4 lists the social support related activities that were offered by SF as part of its pandemic response.

How it all started: “Together our strength can become the true capacity amidst national crises”

**Table 4:** SF’s COVID-19 response: activities related to social support

Social Support	Start	End
Market Linkage		
Connecting 1800 rural farmers to the local market amidst COVID-19	April 2020	June 2020
Food Pack Distribution		
Distribution of food packages to 78,468 vulnerable households	May 2020	-
Food aid to 24,490 garment workers	June 2020	-
Financial Support		
SF staff donated one day’s worth of salary to the Prime Minister’s COVID-19 fund	April 2020	-
Rescheduled Loan Schemes: the microfinance programme introduced a flexible repayment options and waiver of service charges	October 2020	September 2022
*Promoting Sustainable Health and nutrition Opportunities for Marginalized urban extreme poor population (Proshomon – Mitigation)* provided BDT^a^ 2000 (once) grant to 14,317 beneficiary families living in slums	December 2020	-
ILUEP* Project Grant Distributed to 3,548 beneficiaries	July 2020	October 2020
Education Support		
*Improving Lives of Orphan Children in Destitute (ILOD) provided 100 children education through phone calls*	March 2020	February 2021

^a^1 USD = 86.4 BDT (exchange rate at the time of the study)

* ILUEP - Improving Livelihood for Urban Extreme Poor

To understand SF’s response amidst COVID-19 pandemic, key members of the top management was asked how “*it”* all started. One of the key-informants stated, *“When COVID first appeared in Bangladesh, there was lot of fear around us. What is COVID-19? How does it spread? How to treat COVID-19 cases?”* Another key-informant mentioned that, *“… though we had no previous experience in dealing with such type of an emergency, we had a principle to take action in case of any emergencies.”* One of the key-informants summarised this as, *“Our mission says we will improve quality of life through sustainable and effective interventions but at that time we were not thinking of sustainability, we were concerned about whether it was effective. We focused on effectiveness and for that we exerted a lot of labour.”* Upon further probing, participants mentioned that SF’s response to the COVID-19 pandemic began by identifying the *nature* of the challenge, *what* was needed and *who* needed it. It was decided to prepare for pandemic services based on the organisation’s strengths.

While planning often appears to be one of the first challenges, implementation of the plan, considering the challenges *on the ground* are also critical. After studying SF's entire pandemic response, it was clear that its implementation was the result of a “*blending of strengths”,* learning from the organisation’s own experience of implementation of development initiatives and that of the others, too. The leadership team of SF conducted a series of discussions with their field teams in both rural and urban Bangladesh. As SF covers about half of Bangladesh through various development initiatives, their experience helped in summarising the impending challenges, and identifying people/groups at risk of contracting COVID-19 and facing its consequences across Bangladesh. Based on these discussions, SF embarked on their first health and non-health related responses towards the pandemic as listed in Tables 3 and 4. In the course of time, SF also observed and learned from what other development partners/NGOs were doing and had series of consultations with local administrations and community leaders/elites for an organised response to newer challenges. This was very important in identifying and discarding what appeared to be not working and thereby devising a more precise plan to reach the vulnerable groups. When discussing SF’s response, a member of the senior management stated, *“… after the success of Narayanganj hospital, we gained momentum. This eventually led to expanding COVID hospital services in the border districts.”* Thus, one of the hallmarks of SF’s pandemic response at the beginning was to capitalize on their own and others’ strengths and experiences, including that of the local key persons. It also eventually became the key factor for the leadership to stay informed on the course of the pandemic, especially at the local level, and keep the response evolving accordingly. SF’s pandemic response was thus, in many ways, *bottom up.*

### Fast, Holistic and Dynamic: the breadth and depth of the response

Based on the interviews with SF’s staff, it was apparent that the hallmark of SF’s response to the COVID-19 pandemic was fast, holistic and dynamic. This essentially implies that the SF leadership realised the breadth and depth of the pandemic and related needs. SF identified COVID-19 not only as a threat to population health but to other aspects of lives as well. Therefore, the first challenge for the leadership was to ensure continuation of the ongoing development programmes for which need for information and related awareness to prevent contracting the disease was deemed to be essential. Thus, the initial response was to focus on raising awareness and promoting prevention guidelines as per government’s (and that of other national and international organisations’) suggestion and plan. One of the first teams that started acting on SF’s initial plan was the Home and Community Care Limited (HCCL) which is a programme dedicated to bringing care to older people in the comfort of their own home. One of the senior members of the HCCL team stated: *“It was our first goal to ensure that everyone in the household – the patient, our caregiver, the family – they all knew what COVID was, and they all were determined to follow the guidelines that was set out by the WHO* (World Health Organisation)*.”* This became the cornerstone of continuation of home-based care throughout a global pandemic. However, as the pandemic grew the diverse need for COVID-related healthcare provision became more and more apparent.

The identification of the first case of COVID-19 related death in Bangladesh and the development of the Bangladesh Pandemic Response Plan (BPRP) all happened in March 2020. Hence, March 2020 is very significant in the pandemic history of Bangladesh. As the timeline shows, along with the Government of Bangladesh, the SF management, too, responded very early. The leadership of SF realised new and expanded health provision was required to combat COVID-19 pandemic and all efforts should aim to strengthen the government’s response (to the pandemic). This is further explained in the statement of one of the key-informants (from the management), *“…the Government of Bangladesh was not prepared at all to deal with COVID and the logistical issues that come with it, like personal protective measures, ICU, HDU and various other issues government had no preparation.”* Thus, the core areas of SF’s COVID-related healthcare provision were twofold: making patient care and support available and strengthening the government’s response to the pandemic. As stated by a member of the senior management: *“… to sustain the reputation gained and continue the work for longer period, one needs relationship building and networking with the government.”* This collaboration was made official by signing a memorandum of understanding (MoU) between SF and the Directorate General of Health Services. With the MoU, units dedicated to patients of COVID-19 only were set up in Keraniganj, a sub-district of Dhaka district where the capital is situated and in Narayanganj, a district adjacent to Dhaka. This indicates that SF’s response to the pandemic was not only fast but also structured. Such approach helped SF to make new therapeutics available to the population, such as convalescent plasma therapy at their hospital as early as June 2020. In addition, as indicated in Table 3, SF has also helped in setting up ICU units and training staff to carry out ICU services in government hospitals in Pabna, Meherpur, Chuadanga and Jashore, three of which are border districts of Bangladesh. One of the doctors who led this work described it as *“timely and most effective”*. The border districts were chosen as COVID-19 was spreading across the borders at that time. Eventually SF also set up similar collaboration with the Dhaka South City Corporation (DSCC) to help promote Infection Prevention and Control (IPC) measures and related hygiene behaviour among the citizens under their own Water, Sanitation and Hygiene (WASH) programme.

Along with in-person services, SF recognized the need for accessing healthcare remotely when strict travel restrictions were imposed by the government. It set up a 24/7 hotline service staffed by doctors and created a COVID-19 checker messenger *chatbot.* According to one of the participants, the telemedicine services was particularly helpful in the early days of the pandemic in ensuring access to relevant information. During the pandemic, this service became a popular choice as first point of contact for accessing primary care and for referral services. Another very important realisation was the importance of mental health during such difficult times. This initiated the provision of mental health tele- and web-services. The chief executive officer stated in an article published in Dhaka Tribune, *“it’s important to make mental health services as accessible as possible during an emergency, so that those who are vulnerable are able to seek help easily”* [20]. This summarises SF’s health-related response to the pandemic. According to one of the members of the leadership, from the viewpoint of provision, such mix of basic (COVID-19 unit) and critical healthcare (ICU), remote consultation (telemedicine) and collaboration-based (with the government) health related response was timely, innovative and effective. It was appreciated by the people and the government on several occasions, which led to the partnerships in the border districts.

Alongside the provision of needed healthcare to address the pandemic, SF also recognised the non-health related impact on day-to-day lives of the vulnerable populations. To control the spread of COVID-19, the government enforced lockdown which restricted people’s access to basic services and livelihood. Thus, countries like Bangladesh were facing an emergency which was broader than health. So, the appropriate response called for providing not only just health related services, but more of a holistic understanding of the problem and responding accordingly. Due to SF’s structured approach, it could identify the effect of COVID-19 on employment, the local market, etc., including the general reluctance towards IPC measures. To combat the non-health related challenges, but deeply related to practice of IPC measures, SF responded in the following ways:

A. Ensuring additional support to beneficiaries: This included financial support in the form of rescheduled loan schemes for the borrowers of microcredit beneficiaries, flexible repayment options, waiving of service charge and provision of cash aid. Almost all the field operations made provisions for food vouchers and food bags especially for the vulnerable households.
B. Joining forces with the locals: To keep local livelihood afloat and retain access to basic amenities and groceries, SF set up a distribution chain to connect local producers to the local consumers. Such partnership with the locals was also effective in promoting public hygiene and delivering hygiene-related products. This included installation of 585 pedal-based hygiene stations across 228 microfinance branches of SF and urban slums, public toilets, RMG factories, hospitals, community clinic and other SF branches in Dhaka, Narayanganj and Gazipur city corporations with the help of the local youth and businesses. With the help of out-of-work domestic workers, 27,800 textile masks were produced. About 11,500 slum-based households at Dhaka South City Corporation and almost 11,000 female RMG workers received hygiene kits comprised of masks and bathing soaps. More than 1,600 adolescent girls received hygiene kits comprised of bathing soaps, laundry soaps, sanitary napkins, bleaching powder, detergent powder, water pot and handwash container. About 33,500 surgical masks and 86,550 bottles of hand sanitizers were distributed in the communities in the SF working areas. All these distributions were carried out by SF staff in the field with the help of the local volunteers, elites and colleagues from other NGOs.
C. Promoting new normal lifestyle: This was a specific instruction that came directly from the SF central management. The directive was to guide all the existing programmes to make necessary arrangements to create an enabling environment so that people could continue to live their own lives despite the pandemic-related constraints. For example, since schools were closed due to lockdown, SF’s education programme (part of SF’s urban wellbeing programme) initiated remote learning and provided beneficiaries with necessary equipment such as mobile phones. Teleconsultation ensured remote access to healthcare including mental health for the community as well as SF staff. As described below, SF’s own staff were encouraged to attend office remotely and provided with appropriate internet platforms and internet allowances, as well as access to laptops.

### Resource and task sharing: partnership that worked

As explained before, SF’s pandemic response stemmed from analysing organisational (both SF and others) and community’s strength that helped identify the nature of the needs of the vulnerable groups. It also helped in outlining two other dimensions: resource sharing and stewardship. Given the scale of SF’s pandemic response, accumulation and allocation of financial resources was a major challenge from the very beginning. The interviews with the leadership and frontliners of SF suggest that the organisation took a two-pronged approach from the very beginning: allocating money from the organisation’s own funds, including voluntary contribution by the employees, and collecting money through private donations (both individual and corporate). The latter was made through both, personal and organisational communication. For example, SF reached out to a few corporate offices in Bangladesh using its own network to accumulate funds to access the corporate social responsibility fund of those corresponding organisations.

SAJDIA Foundation also received donations, both in cash and kind, from many organisations. With support from a local pharmaceutical company, SF invested BDT 2.5 crore (USD 0.29 million) in their hospitals for their response to COVID-19. Other companies contributed to the development of a COVID-19 symptom checker messenger chatbot. Through this chatbot, almost 11 thousand people were reached. Other contributions by private corporates included providing equipment to set up handwashing devices in urban poor communities, care kits for frontline health workers, Bilevel Positive Airway Pressure machines to the hospital, ventilator and funds for purchasing PPE during the COVID-19 pandemic.

During the pandemic, SF’s WASH programme was one of the most active programmes on the ground which mostly operated in collaboration with local community and NGOs who were working in the same locations. Locally, they recruited volunteers to promote awareness on hygiene and hand washing behaviour. Local volunteers did what SF employees were unable to do due to mobility restrictions during the lockdown. The local smiths helped WASH build the structures required for the handwashing devices installed by the programme. They also recruited local women to ensure young girls in their community had access to hygiene products for menstruation. Moreover, with their members, SF’s *Amrao Manush* (We too are human) programme was able to create a market linkage between small-scale producers (e.g., farmers) and product traders, processors, retailers, etc. by working with its members. One of the key-informants, who coordinated SF’s activities during COVID-19 pandemic, stated that,

*“We always maintain communication and connection with other organizations. We spoke to them to identify the areas in which they required support. “Where are the marginalized communities?” At that time, we signed an MoU with them. Through the MoU we provided service with organization/them.”*

Internally, the SF COVID-19 response team collaborated with their own microfinance programme to provide similar service to the borrowers and their families e.g., awareness raising, distribution of handwash and other hygiene-related materials, creating market linkages to ensure availability of food locally and supporting the livelihood of the producers, etc. Externally, in collaboration with an international organisation operating in Bangladesh, SF’s WASH programme initiated hygiene programmes among the Ready-Made Garment (RMG) workers in three large garment factories. The initiative served more than a thousand RMG workers in Dhaka South. WASH also played a key role in promoting (and helping with) vaccine registration especially among the vulnerable households and those who needed support with it. In addition, SF identified the importance of mental health and collaborated with a local radio programme to expand their operating hours during the pandemic. The programme is the first of its kind in providing emotional support and suicide prevention helpline in Bangladesh.

Another significant collaboration with a foreign organisation was the ‘Lost Stock’ initiative. This was initiated by SF, with an online shopping platform in the UK, to address the impact of cancellation of large-scale orders at garment factories on its workers. The online shopping platform sold the cancelled clothing items for half price and donated food packages worth USD 10.6 for each RMG worker’s household. This initiative benefited more than 78 thousand households. Food packages were also provided to more than 24 thousand RMG workers through other international collaboration.

For SF, while fund generation was an ongoing process, organisational allocation and contributions by the employees helped initiate its pandemic response activities. As a result, SF could embark on the response to the emergency immediately. This made a real difference in attracting both corporate and individual donors/funders and thus a results-based relationship and mutual trust was established. As the central leadership directly coordinated this, all decisions/allocations could be made based on available resources, necessity and accountability.

### Safety: a key word in SF’s response to the COVID-19 pandemic

A characteristic of SF’s response to the COVID-19 pandemic that became apparent during the interviews was ensuring safety of the workplace and colleagues who were working at the frontline. When probed further to understand the aspiration behind such emphasis on SF’s own staff, it was mentioned several times that the top management considered the safety of their colleagues, especially who were working at the frontline, to be of paramount importance. One of the key-informants stated that, *"… and she (the CEO) was concerned about the organization’s staff. Her objective behind the covid hospital wasn’t just to help people, but also to help staff… Where will my staff go if they are affected by covid-19?”.*

As mentioned before, one of the major strengths of SF’s response was 24/7 alert status of the senior leadership team including its chief executive officer. Globally, safety of the workplace and frontline health workers was a major concern throughout the year 2020 and a good period of 2021. During the periods of lockdown in Bangladesh, many organisations adopted policies for remote working, popularly known as ‘work’ from home’. SF too made provision for remote working as early as March 2020. To enable the employees to be able to work from home, logistics and internet support was provided. In addition, massive overhauling of the information technology was done and one of the popular online office platforms was bought which ensured secured and seamless office work for its workforce. For those who needed to be physically present at the office for specific reasons, a roster was created to ensure that only a limited number of persons would be present at the office at any point of time. As part of compliance to IPC measures, handwashing stations with sanitisers, mandatory wearing of masks, and infrared based sterilisation unit at the entrance were set up. During the interviews for this case study, it was mentioned several times that such quick steps in ensuring safety resulted in boosting the morale of the employees. As one of the key-informants mentioned, *“… our safety was given the topmost priority by the management so that we all could continue our work and contribute to our organisation’s vision in ensuring people’s wellbeing.”* Another key-informant mentioned that, *“Authority (SF) supported us always, in every way. They always looked after our staff. The staff felt reassured. When you receive support from the authority, then coordination is better.”*

SF leadership also took specific initiatives to ensure comfort and safety of its healthcare staff and their families. A two-pronged approach was taken:

A. Personal safety of the healthcare staff: Ensuring personal safety of the healthcare staff, including the frontliners, by making sure that everyone had access to PPEs. While other organisations were suffering from a shortage of PPEs, SF leadership realised the need, identified ways to solve the problem and took specific measures, such as mass production, contacting credible suppliers, etc., to ensure availability of the PPEs for the healthcare staff. This was identified as a major difference between SF’s approach and that of other organisations in enabling healthcare staff to provide services as safely as possible amidst the pandemic. One of the interviewees stated that, *“… the whole country was suffering from lack of PPEs. There were reports of counterfeit products. Finding a credible source to procure PPE was hard. Because of the hard work and direct monitoring by the management, SF employees and the healthcare staff were in a relatively better position to continue to provide services amidst the pandemic.”*
B. Reducing risk of the family members of the healthcare staff: Amidst the pandemic, two groups of SF’s staff were directly involved in providing healthcare: the HCCL caregivers who were responsible for providing care to older persons at their home and the hospital staff including doctors, nurses and other relevant personnel. Ensuring personal safety of the healthcare staff at workplace was one risk group sorted. But there still remained a risk to their family members after returning home from work. At the beginning, many tried to stay away from their families by renting places on their own. Considering strict lockdown and news of continuous rise of cases, COVID-19 was already a stigma. Therefore, as soon as people came to know of someone working in the healthcare sector during COVID-19 pandemic, they were either refused to rent or were driven away if already a tenant. Once again, SF stepped in to deal with their healthcare staff’s accommodation problems. In addition to protective gear of the staff themselves, SF ensured safety of the families of HCCL’s caregivers by providing the staff with separate accommodation, catered food and testing facilities from October to May 2022. The same was also provided for SF’s hospital staff. In this regard, one of the key-informants, who was also a member of the senior management of SF, stated, *“… we ensured highest possible safety for our healthcare workers and highest possible comfort to their family members.”*

Throughout years 2020 and 2021, admitting patients who tested positive for COVID -19 to any hospital was very difficult. There was not enough beds or ICU facilities in the country. As the pandemic grew darker, many employees of SF and their relatives/family members contracted COVID-19. Therefore, ensuring safe work environment or making PPEs available was not enough. The SF leadership made provisions for the employees or their family members/relatives with COVID-19 to get admitted at SF’s Narayanganj and Keraniganj hospital as needed. According to the key-informants, this was very comforting for everyone at SF especially the healthcare staff. Moreover, SF acknowledged the pandemic’s impact on mental health of the staff and began to provide counselling services from September 2020. The staff members of SF reiterated in the interviews that SF’s success was attributed to a motivated and dedicated workforce who were well taken care of by their organisation.

### Organisational adjustment that drove SF’s pandemic response

There is another hallmark feature that played a key role in SF’s response to the COVID-19 pandemic. The world, including Bangladesh and SF, all were facing a completely unforeseen challenge, a health emergency that touched more than health. Its response needed creativity, innovation, *out of the box thinking* and quick decision making. One of the key-informants from the higher management stated that, *“Central to SF’s pandemic response was good leadership which enabled prompt decision making, something which was stressed from the very first meeting in relation to how SF should respond to the challenges of pandemic.”* In order to do so, the SF leadership made some adjustment to their routine administrative procedures. This included altered recruitment policy so that staff could be hired quickly and without bureaucratic delays to render required healthcare service during the pandemic, change in procurement policy which entailed increased cap for buying necessary equipment locally without approval by the head office, etc. These administrative adjustments required a certain level of decentralisation in the decision-making process. To ensure operationalisation of these changes in administrative procedures, the chief executive officer was in constant communication via various dedicated WhatsApp groups. The group conversations were also very helpful in getting relevant and quick information about the spread of the pandemic and its context. While SF’s response to the pandemic was multidimensional, these impromptu adjustments in organisational policies made quick and appropriate response possible. When asked why such changes were made by SF to respond to the pandemic, a member of leadership stated, *“I got a lot of support without any question. We have a lot of protocols and policies – procurement policy, HR policy, a lot of things – I had to break everything. I was brave enough to do so because my CEO allowed me.”*

## Discussion

This case study aimed to describe the philosophy, aspiration and strategy of an NGO in Bangladesh in its response to the COVID-19 pandemic. Based on the findings of this case study, it is evident that the impact of a pandemic is beyond health emergencies and pose multidimensional challenges requiring a comprehensive response plan. This case study shows a way forward for the NGOs in Bangladesh and in other relevant contexts on how to embark on a timely comprehensive response to a pandemic. Based on the findings of the study, a framework of 4Cs is suggested as the basis of a comprehensive response to emergency situations such as pandemic or similar crises.

### 4C Framework: a strategic direction for NGOs to respond to a pandemic

**Fig. 1:**
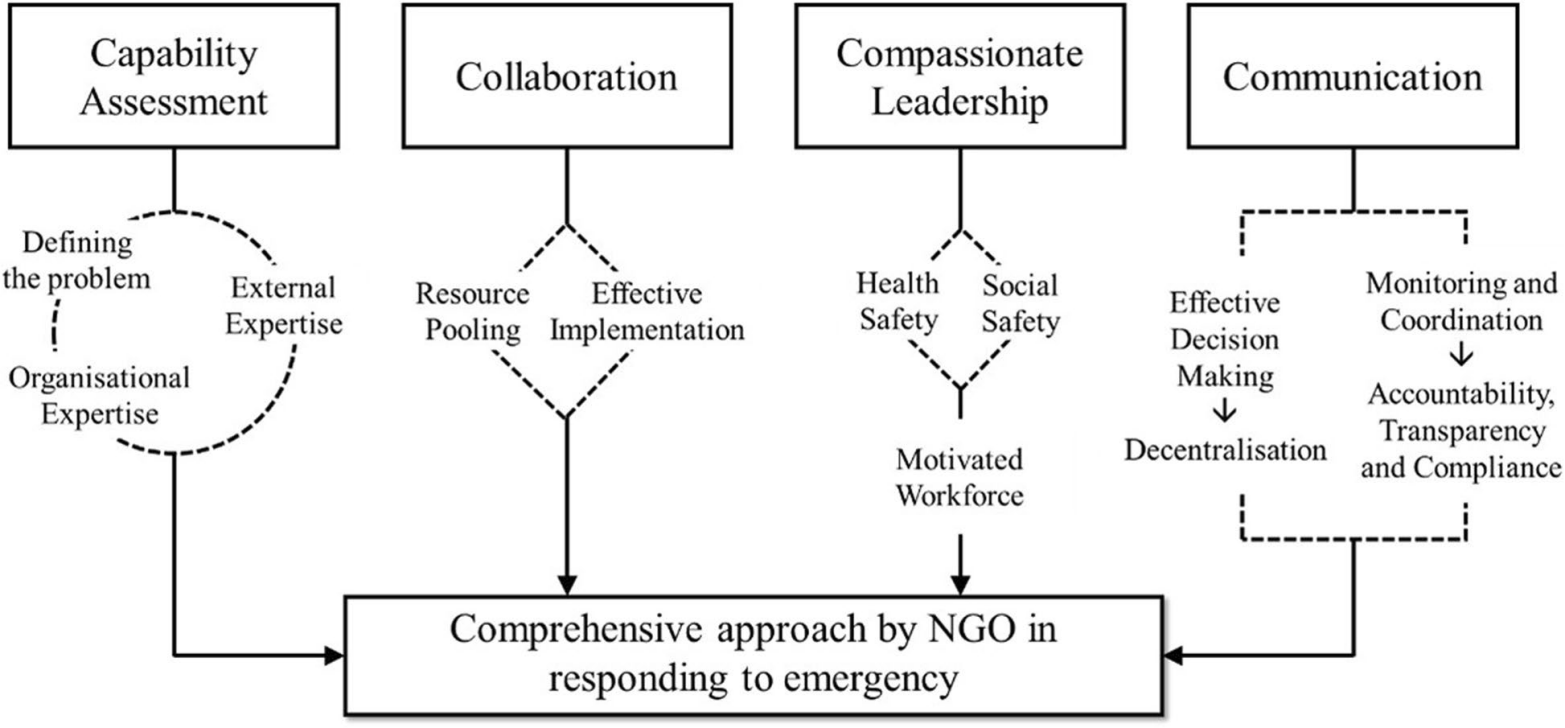
The 4C framework for NGOs to plan and respond to emergency

The findings of the study suggest that pandemic response by an NGO needs to consider two primary objectives. Firstly, to aid the government in responding to the pandemic. Secondly, to adopt a comprehensive response plan in order to address the diverse range of challenges related to the population’s well-being. Such a comprehensive response plan by the organisation in the study included four strategic components. These components can be depicted in a framework of 4Cs as described below (Fig. 1):

- *Capability assessment*: This refers to a full assessment of organisational capability in order to understand the scope of the organisation to initiate a response. As indicated by SF’s experience, this assessment is an ongoing process that starts with a structured approach to define the emergency in three aspects: the *nature* of the problem, *who* (people/group) are in need and *what* response is needed. Once the emergency situation has been defined, it helps in leveraging on the organisation’s own expertise as well as identifying expertise that will be required from outside the organisation in order to ensure the response plan can address multi-dimensional challenges and diverse aspects of people’s wellbeing. Such capability assessment is key to a creative and innovative emergency response which ensures that resources are optimised and appropriately allocated thereby making the best use of its own strength and capacity. Experience on response to COVID-19 in Taiwan suggests such capability assessment in terms of identification of competency, understanding knowledge requirement, defining specific responsibilities and thereby assembling the emergency management team should be a priority during an ongoing emergency to initiate response [21].
- *Collaboration with stakeholders*: The next step in the framework is to initiate strong and structured collaboration with relevant stakeholders. This is to recognise that a comprehensive response to an emergency cannot be done in isolation. It can be a confusing as well as unchartered territory from policy making to implementation which can be eased through effective collaboration among relevant stakeholders to ensure strong response. From SF’s experience it is evident that such collaboration can help in pooling resources (funding and expertise), ensuring effective delivery of the response plan and managing contextual challenges (leveraging on the strength of the local partners and the community) and thereby becoming part of the governance of the emergency response that is stewarded by the government (effective public private partnership). Recent evidence has shown how response to COVID-19 pandemic have failed to devise an effective public private partnership and how crucial it is to have such partnership for emergency management in the context of LMICs [22–24].
- *Compassionate leadership*: One of the major areas of concern in the COVID-19 pandemic has been the safety of the health and care workers. Often referred to as the front-line workers, they have risked their lives everyday by continuing to work even in the darkest periods of the pandemic. There is evidence that these frontline health and care workers not only faced health risks but also faced a range of social and individual risks which impacted on their personal as well as their family’s wellbeing [25–29]. The findings of this case study suggest that this was one of the major concerns for SF too. Hence, their response to COVID-19 specifically focused on ensuring basic safety measures for all its employees and more advanced safety measures for their frontline health and care staff. Additionally, provision of housing, food and support for the frontline workers helped address social and family related risks. The combination of all these support by the organisation played a key role for the SF staff to evolve as a dedicated and motivated workforce in facing the pandemic. Based on the experience of service provision amidst COVID-19 pandemic, several research have described such compassionate leadership as one of the important tools to boost the morale of the health and care staff, thus ensuring continued and quality services [30–32]. Therefore, for any organisation to plan an emergency response, it is of paramount importance to have specific measures in place to address health and social risks of its staff to ensure their personal and their family’s wellbeing.
- *Communication*: This refers to establishing quick and effective communication channel among the different tiers of the organisation, between frontliners and administrative staff including senior management. This enables an organisation to make quick and timely decisions through direct communication channels and make necessary adjustments regarding financial resources, human resources, procurement, etc., while bypassing usual bureaucratic administrative steps. Thus, effective communication is essentially linked to organisational accountability, transparency and compliance with regard to emergency management. Quick and accessible communication channels such as group chats and calls using platforms like WhatsApp can help in creating direct and instant access to the management and receiving important decisions quickly. Such a communication strategy has been described by others as an effective way of decentralisation of the administrative processes as well as a means to monitor the organisation’s activities [33–36]. In addition, as highlighted by SF’s experience, it helps in gaining real time insights of the implementation process of the response plan on the ground by the organisation’s leadership.

### Limitation

The findings reported in this study originates from the experience of an NGO which has considerable presence in Bangladesh. It represents the context of a not-for-profit private sector and not other types of actors in relation to emergency management. Thus, the study cannot call on national action plan based on its findings but can advise similar NGOs in contexts similar to Bangladesh on how to plan and implement emergency response, and contribute to management of future emergencies.

## Conclusion

NGOs play a crucial role in ensuring people’s wellbeing especially in resource poor settings. Bangladesh’s response to the COVID-19 pandemic and related experience is not unique to the country. Many LMICs have resource scarcity and have been forced to adopt a less than optimum response to contain the aftermath of the pandemic. The 4C framework suggested here can be the basis for organisations to embark on a comprehensive response plan to future pandemic(s) or similar emergencies in the following ways:

- It can be helpful for the policy makers, especially the government, of LMICs to provide stewardship by collaborating with NGOs.
- In can be especially helpful for the NGOs in resource poor settings in pooling and sharing resources and forging effective and efficient collaboration(s).
- It can be a strategic guidance for the policymakers, governments and NGOs to plan emergency response in quickest possible time and contribute to the national plan in combating similar threats in the future.

## Data Availability

The data and related materials supporting this study’s findings are available from the corresponding author upon reasonable request.
